# Plasma metabolomic signatures of dual decline in memory and gait in older adults

**DOI:** 10.1007/s11357-023-00792-8

**Published:** 2023-04-13

**Authors:** Qu Tian, Michelle D. Shardell, Pei-Lun Kuo, Toshiko Tanaka, Eleanor M. Simonsick, Ruin Moaddel, Susan M. Resnick, Luigi Ferrucci

**Affiliations:** 1 Translational Gerontology Branch, National Institute On Aging, 251 Bayview Blvd., Suite 100, Room 04B316, Baltimore, MD 21224 USA; 2 University of Maryland School of Medicine, Baltimore, MD USA; 3 Laboratory of Clinical Investigation, National Institute on Aging, Baltimore, MD USA; 4 Laboratory of Behavioral Neuroscience, National Institute on Aging, Baltimore, MD USA

**Keywords:** Dual decline, Memory decline, Gait decline, Metabolomics, Dementia risk

## Abstract

Older adults experiencing dual decline in memory and gait have greater dementia risk than those with memory or gait decline only, but mechanisms are unknown. Dual decline may indicate specific pathophysiological pathways to dementia which can be reflected by circulating metabolites. We compared longitudinal changes in plasma metabolite biomarkers of older adults with and without dual decline in the Baltimore Longitudinal Study of Aging (BLSA). Participants were grouped into 4 phenotypes based on annual rates of decline in verbal memory and gait speed: no decline in memory or gait, memory decline only, gait decline only, and dual decline. Repeated measures of plasma metabolomics were measured by biocrates p500 kit during the same time of memory and gait assessments. In BLSA, 18 metabolites differed across groups (*q*-value < 0.05). Metabolites differentially abundant were enriched for lysophosphatidylcholines (lysoPC C18:0,C16:0,C17:0,C18:1,C18:2), ceramides (d18:2/24:0,d16:1/24:0,d16:1/23:0), and amino acids (glycine) classes. Compared to no decline, the dual decline group showed greater declines in lysoPC C18:0, homoarginine synthesis, and the metabolite module containing mostly triglycerides, and showed a greater increase in indoleamine 2,3-dioxygenase (IDO) activity. Metabolites distinguishing dual decline and no decline groups were implicated in metabolic pathways of the aminoacyl-tRNA biosynthesis, valine, leucine and isoleucine biosynthesis, histidine metabolism, and sphingolipid metabolism. Older adults with dual decline exhibit the most extensive alterations in metabolic profiling of lysoPCs, ceramides, IDO activity, and homoarginine synthesis. Alterations in these metabolites may indicate mitochondrial dysfunction, compromised immunity, and elevated burden of cardiovascular and kidney pathology.

**Supplementary information:**

The online version contains supplementary material available at 10.1007/s11357-023-00792-8.

## Introduction

For over 2 decades, amyloid deposition in the brain has been hypothesized as a cause of Alzheimer’s disease (AD), the most common type of dementia. However, clinical trials targeting amyloid alone have not provided conclusive evidence that such treatments delay or prevent AD [1]. Thus, there is a strong need for discovery studies that can point to novel pathways to Alzheimer’s disease and related dementias (ADRD) for new preventive and therapeutic interventions. The AD Summit 2018 called for novel mechanistic perspectives into systems biology [2].

One early sign of preclinical AD is poor motor function, such as slow gait speed [3]. Slow gait has been observed as early as 12 years prior to symptom onset of mild cognitive impairment (MCI), a transitional stage from normal aging to AD [4]. Recent studies from our group and others have shown that older adults with accelerated declines in memory and gait have higher risk of developing dementia than those with memory or gait decline only [5, 6]. Why those with dual decline show the highest AD risk is not clear.

We hypothesize that older adults who develop both cognitive and mobility problems in the clinical course of ADRD represent an important subgroup with specific pathophysiological characteristics [7]. We hypothesize that this subgroup exhibits specific metabolic alterations that cause dual impairment in cognition and mobility, or pathologies of the central nervous system and other systems negatively affect dual impairment. Metabolomics provides quantitative information on many small molecules in biological fluids and tissues [8]. Characterizing the metabolomic profiles of individuals at high risk of developing ADRD may provide meaningful insights into biological pathways that are altered early in the clinical course and thus help identify individuals most likely to benefit from preventive interventions. Previous metabolomics studies suggest that metabolic dysregulation may be a sensitive, early marker of AD, but such evidence comes from studies with relatively small sample sizes, and findings have not been consistent or robust enough for clinical utilization [9, 10]. One of the more consistent findings is that lower plasma lysophosphatidylcholine (lysoPC) C18:2 is associated with both gait decline and the development of memory impairment [11, 12]. However, none of the previous studies examined shared metabolites associated with both gait and cognitive decline in the same individuals. Our recent systematic review shows that metabolites associated with both memory and gait impairments are implicated in the sphingolipid metabolism pathway [13].

In this study, we aimed to examine metabolomic signatures of dual decline in gait and memory using data from the Baltimore Longitudinal Study of Aging. Based on our research and extensive data from the literature, we hypothesize that older adults who experience dual decline have specific metabolomic changes, particularly in circulating phospholipids.

## Methods

### Study population

We conducted the analysis in the Baltimore Longitudinal Study of Aging (BLSA). BLSA is a prospective study with continuous enrollment since 1958 [14, 15]. Follow-up visits occur every 4 years for participants aged < 60, every 2 years for ages 60–79, and annually for ages 80 and older. The National Institutes of Health Institutional Review Board approved the BLSA protocol. All participants provided written consent at each visit.

Participants were included for analysis if they had: (1) repeated and concurrent data on plasma metabolomics, memory performance, and gait speed in the absence of dementia, (2) no missing baseline covariates, (3) baseline gait speed at least 0.6 m/sec, (4) baseline age 50 + (BLSA sample only). For this study, “baseline” was referred to as the first concurrent assessment of plasma metabolomics, memory, and gait. The final analytical dataset included 2351 person-visits of measured metabolites among 855 BLSA participants.

### Phenotypic groups of dual decline and others

As described previously, we first computed annual rates of change in memory and gait speed using simple linear regression [6]. In the BLSA, usual gait speed was measured over 6 m. Verbal memory was assessed using the California Verbal Learning Test (CVLT) immediate recall. CVLT is widely used to assess verbal memory in adults [16]. We then defined 4 phenotypic groups using cut-points of the lowest tertile of annual changes in memory and gait (CVLT decline of 0.67 points/year, gait decline of 0.022 m/sec/year). The “dual decline” group were those in the lowest tertile of memory decline and gait decline. The “no decline” group were those in the middle and upper tertiles of annual decline in memory and gait. The “memory decline only” group were those in the middle and upper tertile of gait decline but the lowest tertile of memory decline. The “gait decline only” group were those in the middle and upper tertile of memory decline but the lowest tertile of gait decline. For participants who developed dementia, data points of memory and gait at and after dementia diagnosis were not used to define phenotypic groups because we focused on memory and gait decline prior to diagnosis.

### Collection of plasma and measurement of metabolomics

Fasting EDTA plasma samples were collected between January 2006 and October 2018 in the BLSA and frozen at − 80 °C. Metabolomics was analyzed between September and December 2019 via the MxP Quant 500 kit (biocrates life sciences ag, Innsbruck, Austria). Metabolomics was assessed using liquid chromatography with tandem mass spectrometry for small molecules, and lipids and hexoses were measured by flow injection analysis-tandem mass spectrometry. Metabolites with greater than 20% values below the limit of detection (LOD) were excluded. For the remaining metabolites, values below LOD were imputed using the logspline density approach [17]. We also calculated 9 specific ratio measures based on individual metabolites, including the Fischer Ratio (branched-chain amino acids/aromatic amino acids), Global Arginine Bioavailability Ratio (GABR = Arg/(Ornithine + Cit)), Homoarginine Synthesis (hArg/(Arg + Lys), Homocysteine Synthesis (HCys/Met), Hippuric Acid Synthesis (HipAcid/Gly), Indoleamine 2,3-Dioxygenase Activity (IDO = Kynurenine/Trp), Ratio of DHA to EPA, Ratio of Proline to Citrulline, and Sarcosine Synthesis from Glycine (= Sarcosine/Gly) (Table S1).

### Statistical analysis

To examine metabolomic signatures of dual decline, metabolites were first log2 transformed, and then linear mixed-effects (LME) models were used to regress each metabolite on the phenotypic group, time, group-by-time interactions after adjusting for baseline age, sex, race, education, apolipoprotein E ε4 carrier status, and baseline gait speed and memory. We additionally adjusted for calendar year because participants’ baselines vary. In LME, the most recent metabolomics assessment was the anchor point (time 0). Data points prior were time in years before time 0. For discovery, *p*-values were adjusted for multiple comparisons using the Benjamini–Hochberg approach [18]. Based on results from LME models, we performed class enrichment analyses [19]. In the analysis of 9 metabolite ratio measures, significance was set at *p* < 0.05.

Focusing on metabolite differences between dual decline and no decline, we performed KEGG pathway analysis via https://www.metaboanalyst.ca/. First, we identified IDs from the Human Metabolome Database (HMDB https://hmdb.ca/) for metabolites that showed significant longitudinal differences between dual decline and no decline groups at *p* < 0.05. We then entered HMDB IDs into the KEGG database and reported pathways that were significant at *p* < 0.05.

Because many metabolites were highly intercorrelated, we performed a network analysis to identify metabolite modules. First, we computed Spearman correlations between metabolites and performed weighted gene co-expression network analysis (WGCNA) with hierarchical clustering to identify metabolite modules [20]. Each module was defined by the hub metabolite with the highest intra-module connectivity. Second, we performed principal components analysis and computed the first principal component score for each module. We then used the LME principal component scores to examine module differences between groups.

## Results

Four hundred sixty-one metabolites that had less than 20% values below the limit of detection (LOD) were included for analysis. Table 1 shows the baseline participants’ characteristics. BLSA participants had on average 2.7 metabolomics assessments over a median 7.1 years (up to 12.2 years).

**Table 1:** Baseline participants’ characteristics

	BLSA			
	No decline (*n* = 406)	Memory decline only (*n* = 169)	Gait decline only (*n* = 167)	Dual decline (*n* = 115)
Mean ± SD or *N* (%)				
Age, years	66.6 ± 8.9	70.4 ± 9.2	72.1 ± 9.7	76.6 ± 10.0
Men, *N* (%)	177 (43.6)	77 (45.5)	88 (52.7)	58 (50.4)
Black, *N* (%)	118 (29.1)	51 (30.2)	39 (23.4)	13 (11.3)
Education, years	17.6 ± 2.5	17.6 ± 2.8	17.5 ± 2.9	17.5 ± 2.7
Apolipoprotein E ε4 carriers, *N* (%)	93 (22.9)	51 (30.2)	49 (29.3)	24 (20.9)
Usual gait speed, m/sec	1.17 ± 0.19	1.14 ± 0.20	1.24 ± 0.22	1.16 ± 0.23
Verbal memory	51.9 ± 11.9	54.6 ± 11.3	48.0 ± 11.8	53.5 ± 11.5

Verbal memory was measured using California Verbal Learning Test immediate recall total scores in the BLSA (range 0 to 80)

### Metabolite differences between groups and class enrichment analysis

**Fig. 1:**
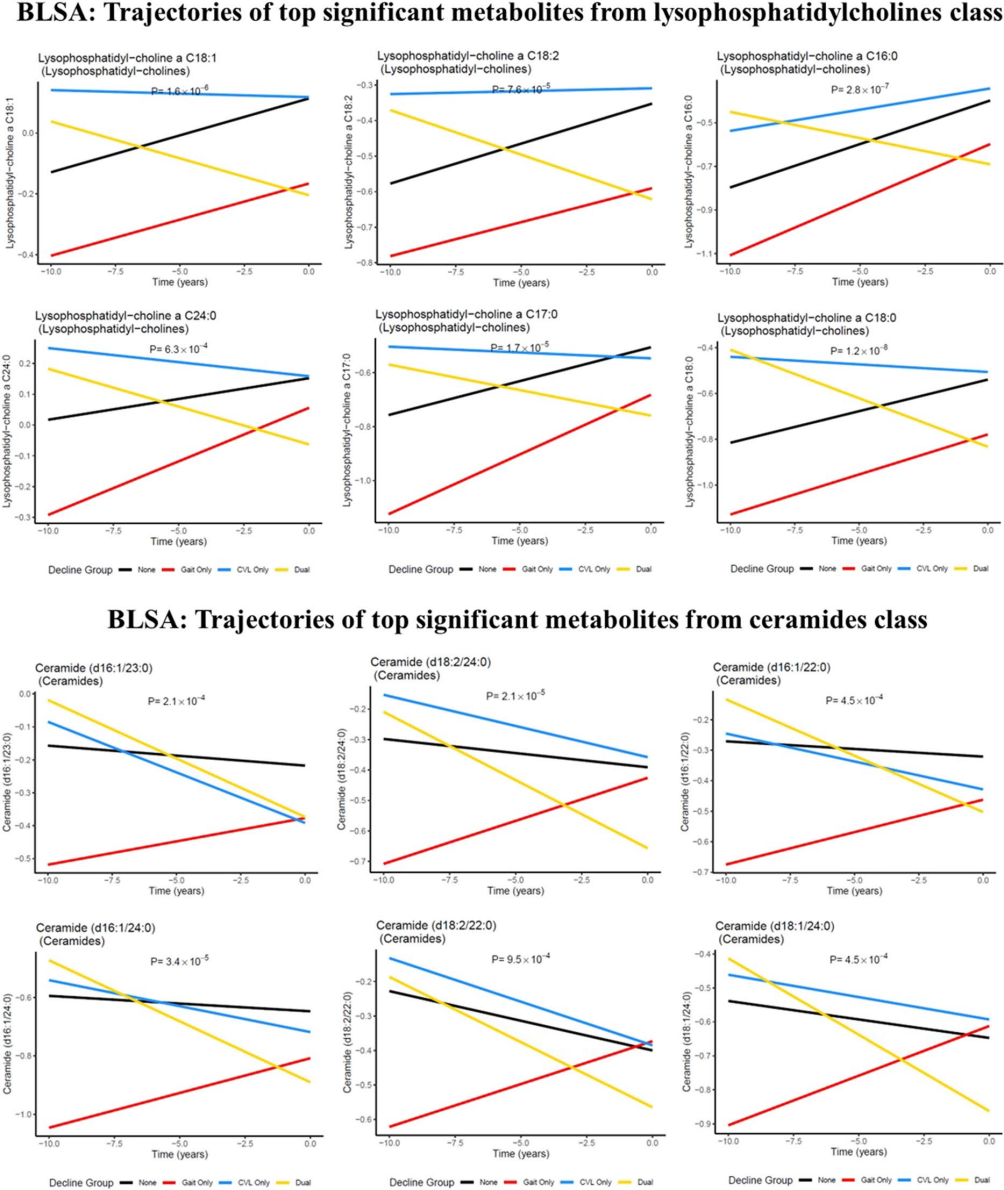
Trajectories of top significant metabolites from the lysophosphatidylcholines (**A**) and ceramides classes (**B**). Legend: *p*-values indicate the significance for group differences, including both cross-sectional and longitudinal comparisons

In the BLSA, 18 metabolites significantly differed among the four groups at *q* < 0.05, including cross-sectional and longitudinal differences (Table 2). These metabolites were from four classes: nucleobases (hypoxanthine), lysoPCs (C18:0,C16:0,C17:0,C18:1,C18:2), ceramides (d16:1/24:0,d18:2/24:0,d16:1/23:0,d18:1/24:0), phosphatidylcholines (PC aa C32:2,ae C44:3,ae C40:1,ae C42:1), and amino acids (glycine). LysoPCs, ceramides, and amino acids classes were significantly enriched (Table 3). Top significant metabolites from lysoPCs and ceramides by groups are shown in Fig. 1 parts A and B.

**Table 2:** Individual metabolites that significantly differed among the four phenotypic groups

Rank	Metabolite names	*p*-value	*q*-value
1	Lysophosphatidylcholine a C18:0	1.19E-08	5.48E-06
2	Hypoxanthine	9.03E-08	2.08E-05
3	Lysophosphatidylcholine a C16:0	2.75E-07	4.23E-05
4	Lysophosphatidylcholine a C18:1	1.56E-06	0.000180053
5	Lysophosphatidylcholine a C17:0	1.71E-05	0.001580532
6	Ceramide (d18:2/24:0)	2.14E-05	0.001643773
7	Ceramide (d16:1/24:0)	3.39E-05	0.002231623
8	Lysophosphatidylcholine a C18:2	7.64E-05	0.004403035
9	Phosphatidylcholine aa C32:2	0.000143795	0.007365506
10	Ceramide (d16:1/23:0)	0.000210124	0.009686713
11	Ceramide (d18:1/24:0)	0.000452942	0.017414705
12	Ceramide (d16:1/22:0)	0.000453311	0.017414705
13	Lysophosphatidylcholine a C24:0	0.000632704	0.02099238
14	Glycine	0.000637513	0.02099238
15	Phosphatidylcholine ae C44:3	0.000728012	0.022374244
16	Ceramide (d18:2/22:0)	0.000949566	0.027359369
17	Phosphatidylcholine ae C40:1	0.001443081	0.03913296
18	Phosphatidylcholine ae C42:1	0.001912612	0.048984116

**Table 3:** Class enrichment analysis results

Rank	Classes	*p*-value
1	Lysophosphatidylcholines	2.35E-07
2	Ceramides	0.00287
3	Amino acids	0.036669
4	Cholesteryl esters	0.064234
5	Hormones and related	0.224229
6	Triglycerides	0.241675
7	Indoles and derivatives	0.43353
8	Carboxylic acids	0.521671
9	Amino acid related	0.579229
10	Dihexosylceramides	0.850643
11	Sphingomyelins	0.858738
12	Phosphatidylcholines	0.883157
13	Dihydroceramides	0.885861
14	Trihexosylceramides	0.888154
15	Fatty acids	0.91344
16	Diglycerides	0.93361
17	Acylcarnitines	0.970894
18	Hexosylceramides	0.979744
19	Biogenic amines	0.991852
20	Bile acids	0.993383

Classes that only included 1 metabolite cannot be estimated for enrichment analysis and are not included in this table. These classes are amine oxides, vitamins and cofactors, alkaloids, carbohydrates and related, cresols, and nucleobases and related

For longitudinal metabolite changes, pairwise comparisons between no decline and declining groups are presented in Figure S1. Compared to no decline, the memory decline only group had significant changes in 23 metabolites at *p* < 0.05 but none were significant after adjustment for multiple comparisons (*q* ≥ 0.05). The gait decline only group had significant changes in 120 metabolites at *p* < 0.05 and PC aa C32:2 remained significant after adjustment for multiple comparisons (*q* < 0.05). The dual decline group had significant changes in 138 metabolites at *p* < 0.05. LysoPCs C16:0, C18:1, and C18:2 were among the top 10 significant metabolites at *p* < 0.05, and lysoPC C18:0 remained significant after adjustment for multiple comparisons (*q* < 0.05). LysoPCs, triglycerides (TGs), amino acids, and cholesteryl esters were significantly enriched.

### Metabolite ratio measures between groups

**Fig. 2:**
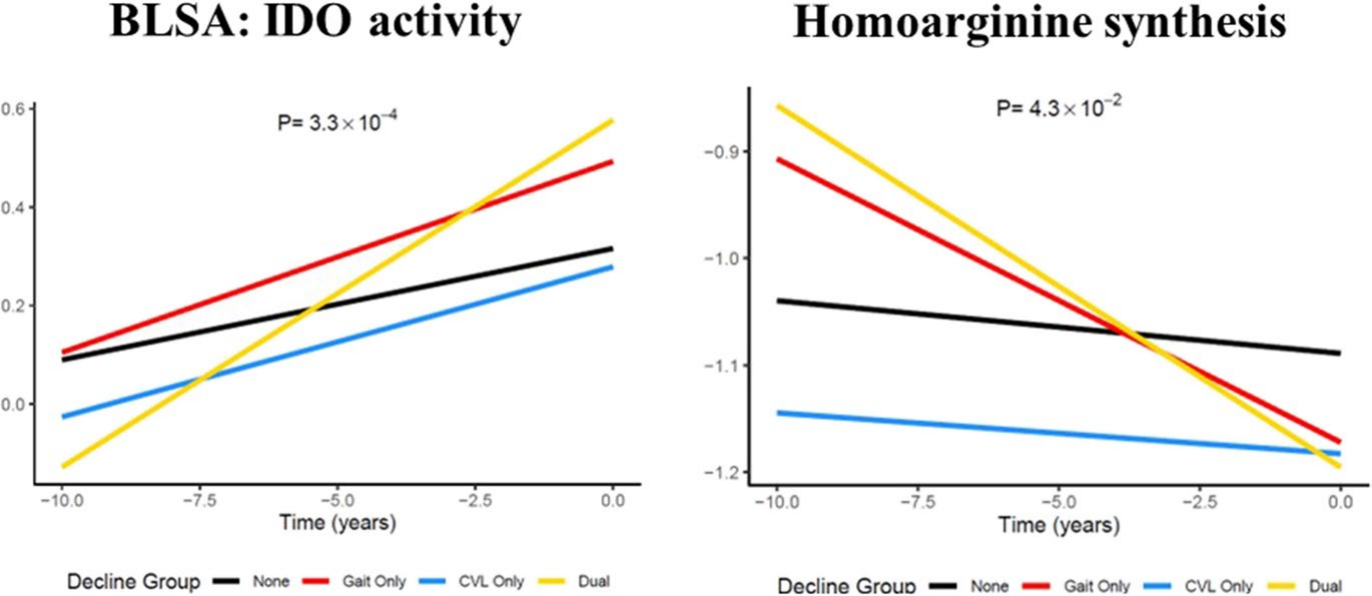
Trajectories of IDO activity and homoarginine synthesis by groups. Legend: *p*-values indicate the significance for group differences, including both cross-sectional and longitudinal comparisons. The dual decline group had a significant increase in IDO activity and a significant decrease in homoarginine synthesis compared to no decline (*p* = 2.72E-05, and *p* = 0.012, respectively)

In the BLSA, IDO activity and hArg synthesis showed significant group differences (*p* = 0.0003 and *p* = 0.043, respectively). Compared to no decline, the dual decline group had a significant increase in IDO activity and a significant decline in hArg synthesis (IDO: β = 0.048, *p* = 2.72E-05; hArg: β =  − 0.029, *p* = 0.012) (Fig. 2).

### Pathway enrichment analysis

**Fig. 3:**
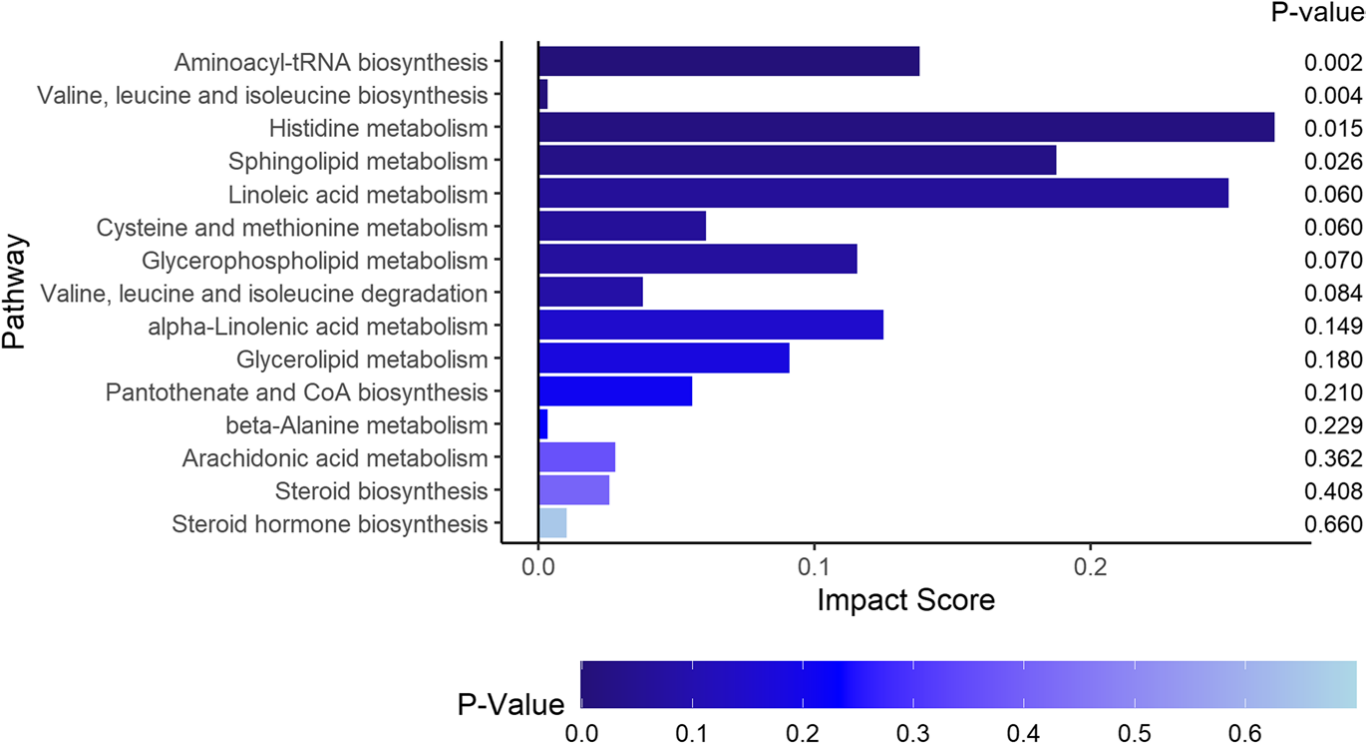
Pathway enrichment analysis for metabolites different between dual decline and no decline groups in the BLSA

For metabolites that differed over time between dual decline and no decline groups, four pathways were significantly enriched in the BLSA, including aminoacyl-tRNA biosynthesis, valine, leucine and isoleucine biosynthesis, histidine metabolism, and sphingolipid metabolism (Fig. 3).

### Weighted correlation network analysis

Network analysis identified 3 modules in the BLSA (Figure S2). Module 1 consisted of mostly TGs, with TG 18:3_34:0 as the hub metabolite. Module 2 consisted of mostly sphingomyelins and phosphatidylcholines with sphingomyelin C16:0 as the hub metabolite. Module 3 consisted of mostly phosphatidylcholines and some amino acids with PC ae C38:0 as the hub. In the BLSA, the dual decline group showed a significant decrease in module 1 (mostly TGs) (β =  − 0.029, *p* = 0.034) but not in module 2 (β =  − 0.017, *p* = 0.26) compared to the no decline grou.

## Discussion

Using longitudinal data on plasma metabolomics collected serially in BLSA participants, we identified specific metabolomic signatures of dual decline in memory and gait, a phenotype that has been previously associated with high risk of dementia. The main findings point to the importance of lysoPCs, ceramides, IDO activity, and hArg synthesis as underlying pathology leading to dual decline in memory and gait. Several metabolic pathways are implicated in dual decline in comparison to no decline, including the aminoacyl-tRNA biosynthesis, valine, leucine and isoleucine biosynthesis, histidine metabolism, and sphingolipid metabolism.

The specific metabolomic signatures of dual decline include metabolites from lysoPCs, TGs, amino acids, and cholesteryl esters, of which lysoPC C18:0 showed the strongest difference and persisted after adjustment for multiple comparisons. It is worth noting that top 10 metabolomic signatures also included lysoPC C16:0, C18:1, and C18:2. These signatures are specific to dual decline, as they were not found in the memory or gait decline only groups. LysoPCs are key precursors of cardiolipin biosynthesis. Cardiolipin is a mitochondria-exclusive phospholipid and is mainly located in the inner membrane of mitochondria, shaping the curvature of the mitochondrial cristae. Recent observational studies from our group and others have shown that specific lysoPCs, including C16 and C18, are associated with skeletal muscle mitochondrial function, muscle mass, and quality, as well as cognitive and mobility impairments [21–23]. A recent randomized clinical trial has shown that supplementation of median-chain TGs increases several lysoPCs in plasma, including lysoPC p-18:0, p-18:1, and C16:0, and importantly, the increase in lysoPC p-18 is associated with improved cognition in mild to moderate AD [24].

Findings of ratio measures of IDO activity and hArg synthesis specific to the dual decline group provide additional insights into mechanisms underlying dual decline. An increase in IDO activity may indicate immune dysregulation and inflammation and the IDO/Kynurenine pathway have been implicated in many associated chronic diseases, including disorders in the musculoskeletal system [25–27]. A decrease in the hArg synthesis may indicate elevated burden of cardiovascular and renal pathology [28–30]. Indeed, homoarginine concentrations are associated with both cardiovascular and chronic kidney disease [28, 31].

In addition to individual metabolite analysis, we extend our examination to pathways and metabolite-to-metabolite interactions using a multifaceted approach. The current pathway analysis results are consistent with our recent literature review that the sphingolipid metabolism pathway is implicated in both memory and gait impairments [13].

This study has several strengths. First, the longitudinal assessments of plasma metabolomics and functional outcomes of memory and gait allow us to investigate within-individual trajectories of metabolites over time underlying dual decline in gait and memory. Second, in addition to the analysis of individual metabolites, we performed enrichment analysis, pathway analysis, and weighted correlation network analysis, which provide additional insights into mechanisms based on metabolite-metabolite interactions. Third, the metabolite ratio measures, such as hArg synthesis and IDO activity, provide information on metabolite functions within the context of physiological systems and health-related conditions. Further, the study population is diverse, including Black and White participants and both men and women, with proportions similar to those of older population in the USA. The depth and richness of the phenotypic characterization and repeated plasma metabolomics provide the first empirical evidence on metabolomic signatures of dual decline in aging.

In conclusion, older adults who experience dual decline have the most extensive alterations in lysoPCs, ceramides, IDO activity, and hArg synthesis. Longitudinal metabolite patterns of dual decline may indicate mitochondrial dysfunction, compromised immunity, and increased risks of cardiovascular and kidney diseases. Our findings provide insights into potential mechanisms underlying dual decline and high dementia risk [6, 32]. Future dementia prevention and intervention strategies may aim to preserve mitochondrial function, maintain healthy immunity, and reducing risks of cardiovascular and kidney diseases, especially in older adults who experience dual decline of memory and gait speed.

### Supplementary information

Below is the link to the electronic supplementary material.

Supplementary file1 (DOCX 299 KB)

## Data Availability

Individual participant data that underlie the results reported in this article, after de-identification (text, tables, figures, and appendices) will be available upon request. Scientists interested in accessing the data should access the website https://www.blsa.nih.gov/ and submit a methodologically sound proposal to the BLSA committee.
