# Supplementary material for: Plasma metabolomic signatures of dual decline in memory and gait in older adults
Source: GeroScience. 2023 Apr 13;45(4):2659–67. doi: 10.1007/s11357-023-00792-8 (PMC10651620; doi:10.1007/s11357-023-00792-8)
Supplement: Supplementary file 1 — Supplementary file1 (DOCX 299 KB) [file 11357_2023_792_MOESM1_ESM.docx]

**Table S1: Definitions for metabolite ratio measures**

**Figure S1: Volcano plots of top significant metabolites that showed longitudinal differences between each declining group and the no decline group.**

**Figure S2: Weighted network analysis**

| Short Name | Name | Formula |
| --- | --- | --- |
| Fischer Ratio | Fischer Ratio | (Ile + Leu + Val) / (Phe + Trp + Tyr) |
| GABR | Global Arginine Bioavailability Ratio | Arg / (Orn + Cit) |
| HArg Synthesis | Homoarginine Synthesis | HArg / (Arg + Lys) |
| HCys Synthesis | Homocysteine Synthesis | HCys / Met |
| HipAcid Synthesis | Hippuric Acid Synthesis | HipAcid / Gly |
| IDO Activity | Indoleamine 2,3-Dioxygenase Activity | Kynurenine / Trp |
| Ratio of DHA to EPA | Ratio of Docosahexaenoic Acid to Eicosapentaenoic Acid | DHA / EPA |
| Ratio of Pro to Cit | Ratio of Proline to Citrulline | Pro / Cit |
| Sarcosine Synthesis from Gly | Sarcosine Synthesis from Glycine | Sarcosine / Gly |

**Table S1. Definitions for metabolite ratio measures**

**
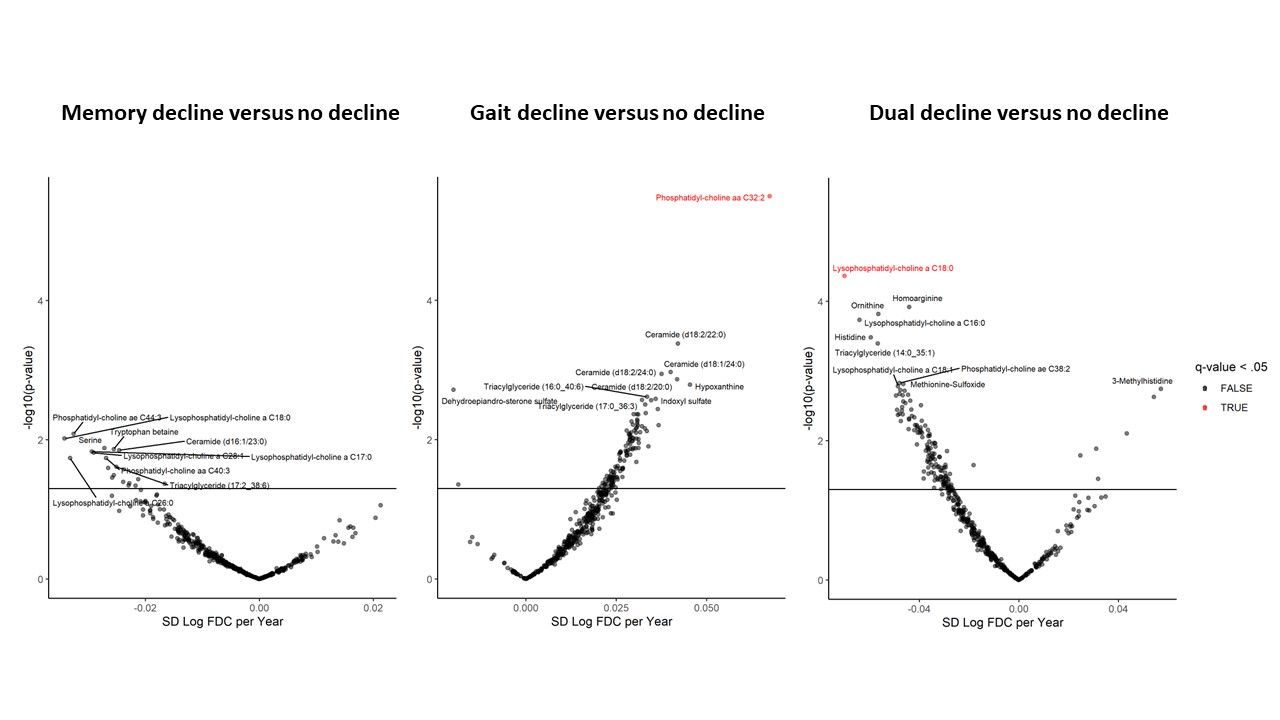
**

**Figure S1. Volcano plots of top significant metabolites that showed longitudinal differences between each declining group and the no decline group.** Legend: top 10 ranked metabolites based on significance is labeled with metabolite names.

**
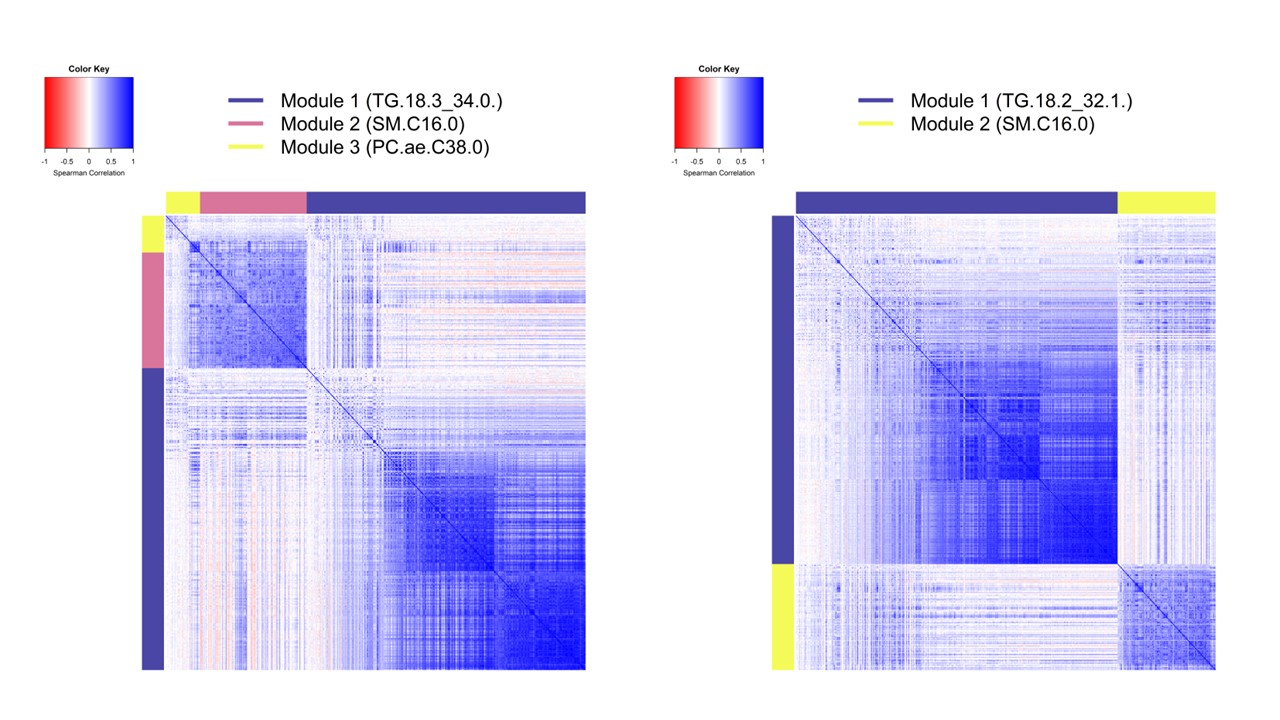
**

**BLSA:**

- Module 1 (303 metabolites): Mostly triglycerides
- Module 2 (116 metabolites): Mostly sphingomyelins and phosphatidylcholines
- Module 3 (37 metabolites): Mostly phosphatidylcholines and some amino acids

**Figure S2. Modules from weighted network analysis.**
